# Towards coherent combining of X-band high power microwaves: phase-locked long pulse radiations by a relativistic triaxial klystron amplifier

**DOI:** 10.1038/srep30657

**Published:** 2016-08-02

**Authors:** Jinchuan Ju, Jun Zhang, Zumin Qi, Jianhua Yang, Ting Shu, Jiande Zhang, Huihuang Zhong

**Affiliations:** 1 College of Optoelectronic Science and Engineering, National University of Defense Technology, Changsha, 410073, China; 2 Northwest Institute of Nuclear Technology, Xi’an, 710024, China

## Abstract

The radio-frequency breakdown due to ultrahigh electric field strength essentially limits power handling capability of an individual high power microwave (HPM) generator, and this issue becomes more challenging for high frequency bands. Coherent power combining therefore provides an alternative approach to achieve an equivalent peak power of the order of ∼100 GW, which consequently provides opportunities to explore microwave related physics at extremes. The triaxial klystron amplifier (TKA) is a promising candidate for coherent power combing in high frequency bands owing to its intrinsic merit of high power capacity, nevertheless phase-locked long pulse radiation from TKA has not yet been obtained experimentally as the coaxial structure of TKA can easily lead to self-excitation of parasitic modes. In this paper, we present investigations into an X-band TKA capable of producing 1.1 GW HPMs with pulse duration of about 103 ns at the frequency of 9.375 GHz in experiment. Furthermore, the shot-to-shot fluctuation standard deviation of the phase shifts between the input and output microwaves is demonstrated to be less than 10°. The reported achievements open up prospects for accomplishing coherent power combining of X-band HPMs in the near future, and might also excite new development interests concerning high frequency TKAs.

Stimulated by the significant progress of modern pulsed power technology pioneered by J.C. Martin and coworkers in 1960s^1^, intense relativistic charged particle beams in nanosecond-scale became available in experiments, which have provided opportunities for many multidisciplinary researches such as, nuclear physics, X-ray flash radiography, Z-pinch, relativistic vacuum electronics, as well as the newly emerged field of bioelectrics^2^. Particularly, as the research focus of relativistic vacuum electronic devices^3^, high power microwave (HPM) generator has gained tremendous progress over the past decades and is still experiencing unprecedented development. The produced HPMs are of great interest for both scientific and civil applications, *e*.*g*. high power radar, power beaming, particle accelerators, plasma heating, and space propulsion, to name but a few^4^. The state-of-the-art HPM generators are able to produce microwaves with peak power in excess of 1 GW over frequencies ranging from P-band to Ku-band, and they are developing towards the directions of high output power, high efficiency, long pulse duration, and repetitive operation^5^. Remarkably, X-band HPMs in 30 Hz output with peak power of 2 GW and pulse duration of more than 100 ns have been demonstrated recently in experiment using an overmoded relativistic Cerenkov generator^6^. Notwithstanding the inspiring achievements, the output microwave power of a single HPM generator can hardly be further enhanced owing to an intrinsic physical limit: surface plasma formation due to breakdown on metal surface induced by ultrahigh radio-frequency electric field strength, usually termed as radio-frequency breakdown^3^,^4^,^5^,^6^,^7^.

Like in fiber optics^8^, a promising development trend of future HPM research is to combine coherently multi HPM generators in order to pursue an equivalent peak power of the order of hundreds of GWs^4^. It is known that coherent power combing requires exactly the same operation frequency and stable phase differences between the individual HPM sources. In terms of HPM oscillators, there have been some experimental demonstrations of coherent combing of Cerenkov superradiances to achieve an equivalent peak power up to ∼10 GW^9^,^10^. Nevertheless, the superradiance sources have to be driven by a common accelerator to realize channel-to-channel synchronization in time, and moreover the radiation pulse duration and energy are far below the requirements of most practical applications. Compared with oscillator, the relativistic klystron amplifier (RKA)^11^,^12^,^13^,^14^, owing to its specific capability to lock the output HPM frequency and phase by input signal, is naturally suitable for coherent power combing. Previous investigations regarding RKAs have demonstrated GW-class long pulse HPM radiations in L-band and S-band^13^,^14^,^15^,^16^,^17^. In order to achieve a higher *Pf*^2^ factor^4^,^7^, scaling RKA to higher frequency bands (such as X-band) leads to the birth of triaxial klystron amplifier (TKA). It is expected that the TKA possesses higher power handling capacity by using coaxial interaction cavities with large radius^18^,^19^,^20^,^21^, where *P* and *f* denote HPM power and frequency, respectively. Nevertheless, the introduction of coaxial structure can easily induce self-excitation of parasitic modes (TEM mode and high order asymmetric TE modes), which could influence beam wave interaction, and result in pulse shortening as well as phase-unlocking. No significant progress therefore has been made in experiment since the proposal of TKA^20^,^21^. Furthermore, by far demonstration of phase-locking of the generated HPMs, the most important characteristic of TKA, is still missing, which somewhat obscures the feasibility of coherent power combining of HPMs in X-band.

Recently GW-class HPM was achieved by developing a multi-beam X-band TKA which adopted spatially separated drift tunnels to suppress self-excitation of parasitic TE modes^22^,^23^. However the entire structure was rather complicated, and the electron beams were subject to plasma instabilities and beam loss during propagation in the small tunnels^24^. Almost simultaneously, we studied theoretically the methods for suppressing TEM mode leakage in order to chase phase-locked long pulse generations by coaxial TKA with large radius^25^,^26^. In this paper, we present investigations into an improved X-band TKA and the associated experimental results of HPM radiations with peak power more than 1 GW, pulse duration in excess of 100 ns, and most remarkably with shot-to-shot phase shift fluctuation (standard deviation) less than 10°. It is moreover found that the experimentally obtained results are in good agreement with those in particle-in-cell (PIC) simulations. In the end, we discuss the issues that should be addressed to achieve a high efficient coherent power combining in experiment.

## Results

### Experimental Results

The proposed TKA (see Methods) was operated on the self-designed HEART-5LT pulsed accelerator (see Methods), which was capable of providing an electrical power up to 5 GW with a full width at half maximum (FWHM) pulse duration of about 150 ns. Closure of the main gas switch of the accelerator was controlled by an external trigger signal, so a small time jitter (standard deviation) of around 5 ns was able to be achieved. The diode voltage could be varied by adjusting trigger time for the main gas switch with respect to that for the transformer primary. An intense electron beam was produced by a knife-edge graphite cathode with an average radius of 4 cm under the mechanism of explosive electron emission^27^. Propagation of the electron beam in the TKA was guided by a longitudinal magnetic field supplied by a solenoid^28^. A small klystron amplifier with an output power of 60 kW was employed as the seed source, whose central frequency and bandwidth were 9.375 GHz and 20 MHz, respectively. Time synchronization of the accelerator, solenoid, and seed source was realized by a digital trigger system. The generated HPM was first converted from TEM mode to TM_01_ mode and then radiated through a horn antenna using a polyethylene disk window with a diameter of 940 mm and a thickness of 70 mm. The TKA was pumped down to a pressure of the order of 10^−3^ Pa in the experiments.

**Figure 1:**
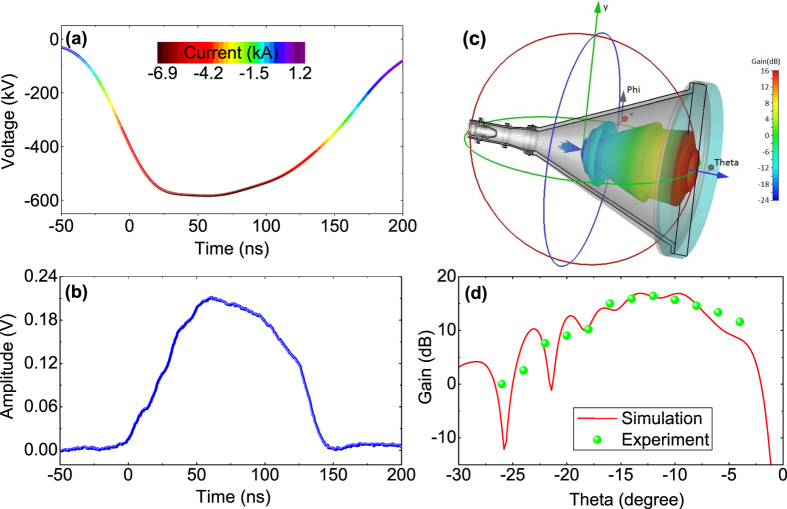
(**a**) Diode voltage evolution recorded in experiment with color indicating the corresponding electron beam current. (**b**) Typical HPM waveform measured by crystal detector in experiment. (**c**) Far field radiation pattern of the antenna for 9.375 GHz microwave obtained by simulation. (**d**) Experimental measurements (dots) together with the simulated far field radiation profile (solid curve).

Typical experimental results are illustrated in Fig. 1 when the guiding magnetic field was approximately 0.6 T. The diode voltage, shown in Fig. 1(a), exhibits a trapezoidal evolution in time, where the curve color indicates the corresponding beam current. As seen, the diode voltage reaches a minimum of about −580 kV at *t* ≃ 50 ns and possesses a FWHM duration of about 153 ns. The beam current shows a minimum of −6.9 kA, which together yields a diode impedance of about 84 Ω. Limited by the hall space, only minus angles could be recorded. Figure 1(b) shows a typical HPM waveform measured in experiment by the crystal detector at the main radiation angle in the horizonal plane. It can be noticed that the HPM radiation also has a quasi-trapezoidal shape with a pulse duration of about 103 ns. After reaching a peak at *t* ≃ 60 ns, the HPM waveform shows a slowly descending flat-top till *t* ≃ 120 ns. Decrease of the HPM amplitude is related to drop of the diode voltage flat-top owing to diode impedance rise, which is induced by cathode plasma expansion during long pulse operation^29^. The total produced HPM power was evaluated to be about 1.1 GW by integrating microwave power densities measured over the angle range *θ* = [−4°, −26°] with a step of 2°. The fraction of radiation power beyond the angle range was too small to be taken into account. Recalling the input microwave power of 60 kW, the designed TKA achieved a gain of 42.6 dB.

**Figure 2:**
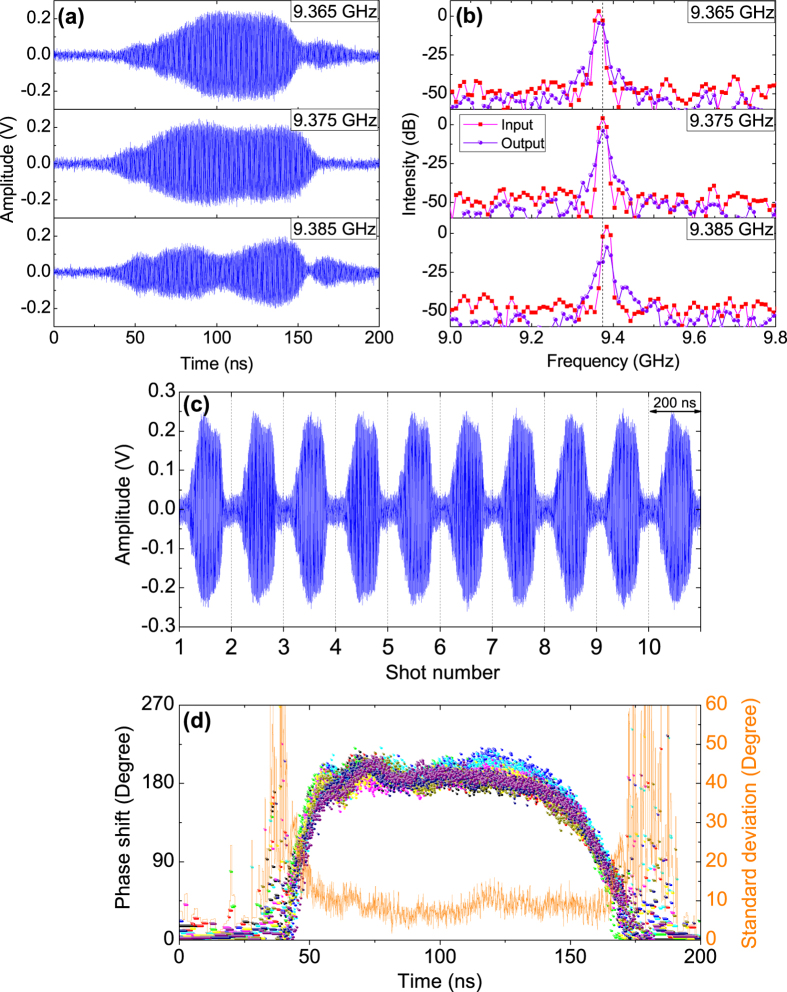
(**a**) Real-time waveforms of output HPMs for three different frequencies *f* = [9.365, 9.375, 9.385] GHz. (**b**) Associated frequency spectra of the input (squares) and output (balls) microwaves respectively, where the dotted line indicates the frequency of 9.375 GHz. (**c**) Sequence display of ten consecutive output HPM waveforms with frequency of 9.375 GHz. (**d**) The corresponding phase shifts between the input and output microwaves for the ten shots shown in (**c**). The right-hand label indicates standard deviation of the ten phase shifts as a function of time.

The output HPM mode was determined by comparing the radiation profiles observed in experiment and simulation, for which the mode converter including the radiation horn were simulated by the electromagnetic software: CST microwave studio. As illustrated in Fig. 1(c), the far field radiation for 9.375 GHz microwave shows a doughnut-shaped pattern with a hollow on the axis. The maximum gain of about 16 dB appears around *θ* = −14°. Uncertainty of the gain measured in experiment is small, because the generated HPM amplitudes are stable as seen in Fig. 2(c). Figure 1(d) shows that the experimental measurements are reasonably consistent with the radiation profile obtained in simulation, which implies pure TEM mode was extracted from the TKA output. It can be noticed in Fig. 1(d) that the experimental results are slightly larger than the simulation predictions for paraxial angles. That is probably because the measurement distance of 6 m does not satisfy the far field requirement: *L*_*ff*_ > 2*D*^2^/*λ*^30^, where *D* and *λ* represent diameter of antenna dielectric window and microwave wavelength, respectively. For the frequency of 9.375 GHz, *λ* = 3.2 cm, along with *D* = 94 cm, yields *L*_*ff*_ ≃ 55 m. So the radiation profile measured in experiment was actually in near field (Fresnel zone) rather than far field (Fraunhofer zone), which leads to the mismatch observed in Fig. 1(d). However, it should be noted that the radiation pattern mismatch does not affect the evaluation of total HPM power.

When the crystal detector was removed, real-time waveforms and phase shifts of the input and output microwaves were recorded by a 20 GHz digital oscilloscope (LeCroy WaveMaster), which allowed to examine frequency-locking and phase-locking capabilities of the TKA. Figure 2(a) shows waveforms of the output HPMs for three frequencies available from the seed source *f* = [9.365, 9.375, 9.385] GHz, respectively. The input microwaves from the seed klystron amplifier can be viewed as continuous electromagnetic waves with stable amplitude and phase. The associated spectra of the input and output microwaves obtained by fast Fourier transform are given in Fig. 2(b), which demonstrate success of frequency-locking. Namely, the output microwaves have exactly the same frequencies as the input ones. It is apparent in Fig. 2(b) that expect the main peak no other frequency peak can be observed in the spectra, suggesting excitation of parasitic TE modes was substantially suppressed in the TKA by the reflectors (see Methods). Furthermore, no output HPM was detected when the input microwave was blocked, which indicates the TKA indeed operated as amplifier without self-oscillation of TEM mode. It is worth mentioning that frequency-locking provides a necessary guarantee for phase-locking, as phase shift between the input and output microwaves is determined by 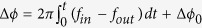, with *f*_*in*_ (*f*_*out*_) and Δ*ϕ*_0_ denoting input (output) microwave frequency and initial phase shift, respectively.

Owing to the TKA was optimized for the frequency of 9.375 GHz, the microwave waveforms of 9.365 GHz and 9.385 GHz shown in Fig. 2(a) are not as good as that of 9.375 GHz in terms of pulse duration, waveform shape, and stability. So the case of 9.375 GHz was further explored for phase-locking. Figure 2(c) shows HPM waveforms measured for ten consecutive shots exhibited in sequence mode. It is shown that the HPM waveforms demonstrate a good shot-to-shot stability. The associated phase shifts Δ*ϕ* between the output HPMs and the input microwaves for the ten shots are given in Fig. 2(d) in overlapped mode. The right-hand label in Fig. 2(d) indicates standard deviation of the ten phase shifts. The phase shift Δ*ϕ* was measured in unit of degree in the range of [0, 360], and Δ*ϕ* = 0 was defined when there was no microwave output. Owing to evolution of the diode voltage and non-infinite *Q* factors of the interaction cavities, initially the frequency of the generated HPM can not be locked exactly to 9.375 GHz. Consequently, the phase shift Δ*ϕ* changes in time. With time increasing, the frequency of 9.375 GHz gradually becomes dominated, so Δ*ϕ* becomes locked to a value around 190° over the time range *t* = [55, 145] ns. When the diode voltage drops, the output HPM tends to cease, and accordingly Δ*ϕ* returns to zero. As shown in Fig. 2(d), phase-locking of the TKA possesses a striking shot-to-shot stability with a fluctuation standard deviation *σ*_*ph*_ less than 10° over main pulse of the generated HPMs. It is furthermore found that *σ*_*ph*_ varies, but not significantly, around 10° for a long operation term.

Higher phase shift fluctuations in onset and termination regions of the HPMs shown in Fig. 2(d) are mainly induced by shift of Δ*ϕ*(*t*) in time due to closure jitter of the accelerator. Fluctuation of the locked-phase is probably caused by shot-to-shot instability of the diode voltage. According to the theory of electron transit time, phase shift fluctuation *δ*(Δ*ϕ*) is correlated to diode voltage *U*_*d*_ in unit of kV by^31^


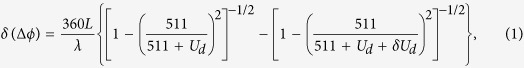


where *L* ≃ 35 cm is the distance from the input cavity to the output cavity, and *δU*_*d*_ denotes diode voltage variance. For the diode voltage *U*_*d*_ = 580 kV, *δ*(Δ*ϕ*)/*δU*_*d*_ is calculated to be approximately 1.1°/kV. In experiments, the diode voltage had a typical shot-to-shot fluctuation standard deviation *δU*_*d*_ ≃ 7 kV, which accordingly leads to a phase fluctuation *δ*(Δ*ϕ*) = 7.7°. The theoretical estimation agrees reasonably with the experimental result *δ*(Δ*ϕ*) < 10°. In this sense, further reduction of the phase shift instability requires sophisticated controls of the diode voltage.

### Results of PIC simulation

**Figure 3:**
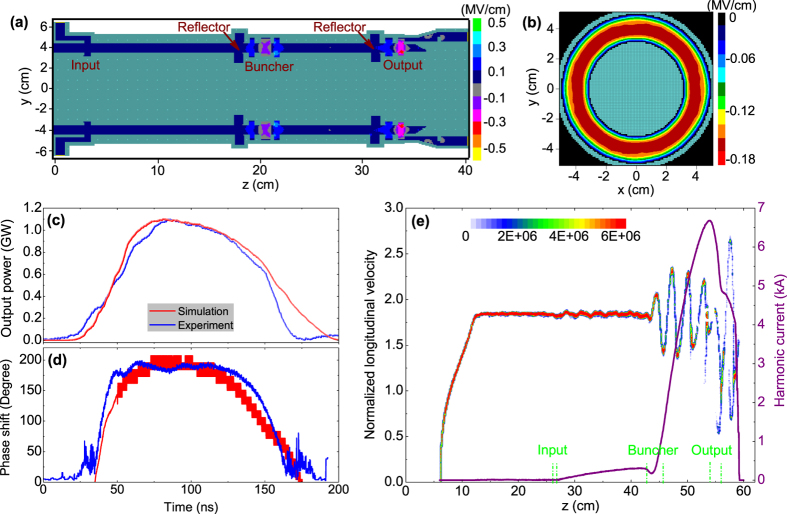
Longitudinal electric field distributions in (**a**) the longitudinal plane and (**b**) the transverse plane located in middle of the buncher cavity. Temporal evolutions of (**c**) the output microwave power and (**d**) the phase shift obtained in simulation (red) and experiment (blue). (**e**) Profile of the harmonic current together with phase space distribution of the modulated electron beam with color weighted by electron number density at *t* ≃ 123 ns when the output microwave power becomes saturated. The green dashed lines indicate longitudinal locations of the input, buncher, and output cavities, respectively.

To take insight into the underlying physics associated with the TKA operation, PIC simulations were carried out with the 3D full electromagnetic code CHIPIC. Because 3D simulation does not allow using fine grid to resolve the cavity chamfers due to a limited total mesh number, and moreover it is rather time-consuming, 3D models were studied as a first step to examine if there existed self-excitation of asymmetric TE modes in the tube. Afterwards, optimization was fulfilled by 2D simulation. The 3D modeling used cartesian coordinates with a grid size of 1 × 1 × 1 mm^3^, and the diode voltage was assigned to be −580 kV with a rise time of 15 ns. Figure 3(a,b) show respectively longitudinal electric field distribution in the longitudinal plane and in the transverse plane located in middle of the buncher cavity obtained in 3D simulation. The time is *t* ≃ 123 ns, when the output microwave becomes saturated. It reveals several important features: (i) both the buncher cavity and the output cavity operates in *π* mode (buncher cavity is with coaxial TM_013_ mode and output cavity is with coaxial TM_012_ mode), which are beneficial to efficient beam wave interaction and HPM generation. (ii) No appreciable electric field exists in the coaxial drift tubes, implying the reflectors successfully eliminate coupling/disturbance between the cavities from downstream to upstream due to TEM mode leakage. (iii) There are no observable electric fields in the reflectors, which therefore would not result in undesired modulation to electron beam. (iv) Symmetric electric field distribution in the transverse plane in the buncher cavity suggests no high order TE mode is excited, so frequency-locking and phase-locking can be guaranteed.

Shown in Fig. 3(c–e) are the results obtained in 2D simulation after careful optimization of the structure with a small grid of 0.5 × 0.5 mm^2^ and a fine grid of 0.05 × 0.05 mm^2^ for the cavity chamfers. Figure 3(c,d) show microwave power and phase shift evolutions in time, respectively, when the diode voltage profile shown in Fig. 1(a) was loaded into the simulation. It can be noticed that both the power and phase shift profiles obtained in simulation agree excellently with the experimental results. Although nearly the same HPM peak power is achieved in experiment and in simulation, experiment requires a higher beam current than simulation, corresponding thus to a lower power conversion efficiency. That is probably because the electron beam quality in experiment based on explosive electron emission is not as good as in simulation. Moreover in Fig. 3(c), compared with the simulated HPM waveform, the experimental one has a slightly shorter pulse duration and a sharper drop slope, which are mainly caused by anode plasma expansion and deterioration of vacuum in the end part of the electrical pulse. In Fig. 3(d), the phase shift Δ*ϕ* measured in experiment exhibits sharper rising and descending slopes than the results of simulation. This phenomenon is caused by a lower *Q* factor of the buncher cavity of about 493 than the theoretical value of 562 due to Ohmic losses on the cavity surfaces. As demonstrated in our previous study^32^, a lower *Q* factor of the buncher cavity requires a shorter transit time to stable phase shift, as saturation time of the cavity shortens with decrease of its *Q* factor. Additionally, in the experimental case, the locked phase has small ripples in its flat-top, which are probably caused by instabilities of the diode voltage, beam current, seed signal, and magnetic field. Plots in Fig. 3(e) reflect beam wave interaction mechanism in the TKA, where the left-hand label represents normalized longitudinal velocity of electron *γv*_*z*_/*c. γ* and *c* denote the relativistic factor and the speed of light in vacuum, respectively. As seen from the phase space distribution, the electrons first get a gentle modulation in velocity by the seed microwave after passing through the input cavity, and then the modulation is significantly strengthened in the buncher cavity. The velocity modulation gradually transfers to density modulation (namely electron bunching) in the coaxial drift tube. Accordingly, the harmonic current *I*_1_ starts from the input cavity and approaches 0.3 kA before the buncher cavity. Before entering the output cavity, *I*_1_ reaches a peak of 6.7 kA, corresponding to a modulation depth of 105%. In the output cavity, most well-bunched electrons lose kinetic energies, and meanwhile the harmonic current decreases, to amplify the local electric field, giving rise to an intense HPM radiation.

## Conclusions and Discussions

In this work, we demonstrate experimental and simulation investigations concerning an improved X-band relativistic TKA, which is capable of producing HPM radiations with peak power of 1.1 GW, pulse duration of about 103 ns, gain of 42.6 dB, and with most importantly phase shift fluctuation (standard deviation) of less than 10°. It is particularly worth pointing out that with the long pulse duration of more than 100 ns, synchronization of the HPMs in time becomes not so sensitive to jitter of the main gas switch closure, so a number of the designed TKAs can be driven by different pulsed power accelerators. With those characteristics, the proposed TKA paves the way for realizing coherent power combining of X-band HPMs in the near future. The presented study would also shed new light and bring out extra opportunities to many related fundamental investigations including space applications of HPM^4^,^33^, microwave discharge-induced plasma physics^34^, modification and measurement of the atmosphere by HPM^35^, and even microwave wakefield acceleration^36^.

One should also keep in mind that despite the striking achievements that we have made, there do exist some features of the TKA which should be enhanced in order to chase a higher equivalent peak power with a practically acceptable combining scale: (i) The output HPM power should be boosted to about 3 GW in a single source to better profit the advantage of high power capacity of coaxial structure. (ii) A higher gain in excess of 50 dB is desired to reduce the input microwave power to less than 30 kW, which could be accomplished by increasing the electron modulation depth using, for example, cascaded buncher cavities. (iii) A better flat-top of the diode voltage and furthermore HPM waveform can be expected by suppressing cathode plasma expansion through coating cesium-iodide (CsI) on the graphite cathode surface^37^. (iv) Last but not least, the shot-to-shot fluctuation of the phase shift needs to be further reduced in order to obtain a higher efficiency of coherent power combining. To give a quantitative impression, supposing the individual TKAs have the same output power, the combining efficiency of *N* units can be estimated by 
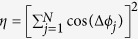
. We could assume that fluctuation of the phase shift Δ*ϕ* obeys normal distribution in experiment, namely 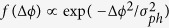. For a scale of *N* = 10 with currently obtained phase shift fluctuation *σ*_*ph*_ ≃ 10°, the mean combining efficiency is 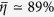. If we look forward to reaching an equivalent peak power of the order of 1 TW, the whole dimension will scale to *N* ≃ 100, for which the phase shift fluctuation should be further reduced to *σ*_*ph*_ < 5° in order to obtain a mean combining efficiency higher than 90%.

## Methods

### Design of the TKA

**Figure 4:**
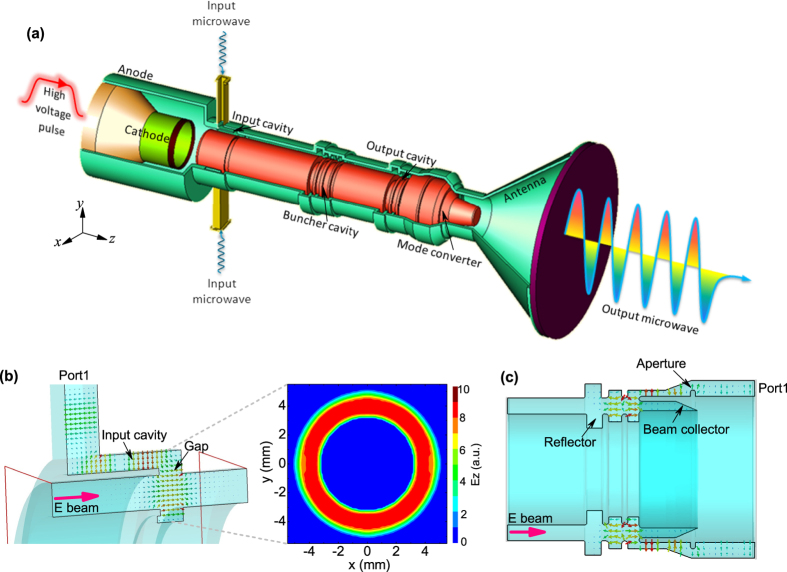
(**a**) Configuration of the proposed X-band TKA, where the high voltage pulse loaded on the diode was provided by the HEART-5LT accelerator, and the input microwave was supplied by the seed klystron amplifier. (**b**) Electric field distribution in the input cavity region, where the right-hand contour plot illustrates magnitude distribution of the longitudinal electric field in middle transverse plane of the cavity gap. (**c**) Electric field distribution in the output cavity region. The pink arrow indicates propagation direction of the electron beam.

As shown in Fig. 4(a), the TKA starts from the diode region including an anode and a cathode (with a knife-edge graphite emitter). The main body is a coaxial structure composed of an input cavity, a bunch cavity, an output cavity, and a mode converter. The cavities take effect to modulate the electron beam, strengthen beam modulation, and extract energy from the bunched electron beam to microwave, respectively. The mode converter is used to convert HPM from TEM mode to TM_01_ mode for radiation. Placed in front of the buncher cavity and the output cavity are two reflectors to suppress leakages of TEM mode and high order asymmetric TE modes^26^. In order to avoid the complexity of axial injection^20^,^21^, input microwave supplied by the seed source is first 50/50 divided and then feed into the input cavity through two lateral rectangular waveguides (standard BJ100). Figure4(b) exhibits field distribution in the input cavity region when 9.375 GHz microwave is injected from port1. As shown, TE_10_ mode in the rectangular waveguide, through TEM mode in the input cavity, converts to coaxial TM_011_ mode in the gap. Along the electron beam propagation path, the electric field in the gap has a dominant longitudinal component, which benefits beam wave interaction. Furthermore, contour plot illustrated in Fig. 4(b) demonstrates high azimuthal uniformity at the electron beam radius of *r* = 4 cm. The external *Q*_*e*_ factor of the input cavity is designed to be around 240, which yields an absorption ratio of 84% for the 6.4 kA electron beam with voltage of 580 kV. The buncher cavity adopts a non-uniform structure operating at coaxial TM_013_ mode with a resonant frequency of 9.42 GHz to strengthen electron beam modulation, mode details of which can be found elsewhere^25^. The output cavity performs at coaxial TM_012_ mode, as indicated by the electric field distribution for the resonant frequency of 9.375 GHz shown in Fig. 4(c). Particularly, the double-gap structure can not only enhance beam wave interaction efficiency but also reduce the maximum surface electric field to below the breakdown threshold of 700 kV/cm for 1.1 GW long pulse operation. A deep beam collector with inclined receiving surface is designed to eliminate/suppress influences of surface heating, secondary electron emission, and anode plasma formation in long pulse microwave generation through the output cavity^7^,^29^. Moreover, external *Q*_*e*_ factor of the output cavity can be adjusted simply by changing the aperture size to optimize HPM extraction efficiency.

### The HEART-5LT accelerator

The HEART-5LT accelerator consists of a pulsed power transformer, a double pulse forming line (PFL)^38^, and a triggered main gas switch. The double PFL is an improved Blumlein structure^1^, which contains two identical separated coaxial single PFLs immersed into transformer oil for insulation. Spiral line is adopted for each single PFL to on one hand increase the PFL impedance to about 60 Ω, and on the other hand lengthen the electrical pulse duration to more than 150 ns. Glycerol with relative permittivity of about 44 is filled into the double PFL as energy storage medium to enhance the breakdown voltage. The double PFL is charged by a high coefficient pulsed transformer with magnetic core, the primary of which uses a 1.7 mF capacitor stock charged to 1800 V. Closure of the transformer primary circuit is controlled by 12 thyristors in parallel. Discharge of the double PFL to diode is determined by closing the main gas switch filled with mixed gases of SF_6_ and N_2_. An all solid-state high voltage generator based on the mechanism of magnetic compression is employed to trigger the main gas switch, so performances of the PFL and TKA can be synchronized in time, and meanwhile the diode voltage can be controlled as expected.

### HPM measurement

The radiated HPM is measured by two receivers (one is fixed as a reference and the other one is shifted) in a fully anechoic chamber at a distance of 6 m from the phase center of the horn antenna. Each receiver consists of a standard antenna with gain of 12.6 dB at the frequency of 9.375 GHz, a waveguide to BNC coupler, and a matching load. A 5 m long microwave cable connects the receiver to 36 dB attenuators together with a crystal detector, whose signal is further transferred by a 30 m long microwave cable to an oscilloscope placed in a shielding room. All the detectors are well covered by microwave absorbing material to prevent electromagnetic noises.

## Additional Information

**How to cite this article**: Ju, J. *et al*. Towards coherent combining of X-band high power microwaves: phase-locked long pulse radiations by a relativistic triaxial klystron amplifier. *Sci. Rep.* **6**, 30657; doi: 10.1038/srep30657 (2016).
